# Identification of G-quadruplex structures that possess transcriptional regulating functions in the *Dele* and *Cdc6* CpG islands

**DOI:** 10.1186/s12867-017-0094-z

**Published:** 2017-06-27

**Authors:** Daniyah H. Bay, Annika Busch, Fred Lisdat, Keisuke Iida, Kazunori Ikebukuro, Kazuo Nagasawa, Isao Karube, Wataru Yoshida

**Affiliations:** 1 School of Bioscience and Biotechnology, Tokyo University of Technology, 1404-1 Katakuramachi, Hachioji, Tokyo 192-0982 Japan; 2 Biology Department, Umm Al-Qura University, Makkah, Kingdom of Saudi Arabia; 3 Biosystems Technology, Institute of Applied Life Sciences, Technical University of Applied Sciences Wildau, Wildau, Germany; 4 Graduate School of Science and Engineering, Saitama University, c/o Saitama Cancer Center, Saitama, Japan; 5 Department of Biotechnology and Life Science, Tokyo University of Agriculture and Technology, Tokyo, Japan

**Keywords:** *Cdc6*, *Dele*, G-quadruplex, Transcriptional regulation, 7OTD

## Abstract

**Background:**

G-quadruplex is a DNA secondary structure that has been shown to play an important role in biological systems. In a previous study, we identified 1998 G-quadruplex-forming sequences using a mouse CpG islands DNA microarray with a fluorescent-labeled G-quadruplex ligand. Among these putative G-quadruplex-forming sequences, G-quadruplex formation was verified for 10 randomly selected sequences by CD spectroscopy and DMS footprinting analysis. In this study, the biological function of the 10 G-quadruplex-forming sequences in the transcriptional regulation has been analyzed using a reporter assay.

**Results:**

When G-quadruplex-forming sequences from the *Dele* and *Cdc6* genes have been cloned in reporter vectors carrying a minimal promoter and the luciferase gene, luciferase expression is activated. This has also been detected in experiments applying a promoterless reporter vector. Mutational analysis reveals that guanine bases, which form the G-tetrads, are important in the activation. In addition, the activation has been found to decrease by the telomestatin derivative L1H1-7OTD which can bind to the G-quadruplex DNA. When *Dele* and *Cdc6* CpG islands, containing the G-quadruplex-forming sequence, have been cloned in the promoterless reporter vector, the luciferase expression is activated. Mutational analysis reveals that the expression level is decreased by mutation on *Dele* G-quadruplex; however, increased by mutation on *Cdc6* G-quadruplex.

**Conclusion:**

*Dele* and *Cdc6* G-quadruplex formation is significant in the transcriptional regulation. *Dele* and *Cdc6* G-quadruplex DNA alone possess enhancer and promotor function. When studied in more complex CpG islands *Dele* G-quadruplex also demonstrates promotor activity, whereas *Cdc6* G-quadruplex may possess a dual function of transcriptional regulation.

**Electronic supplementary material:**

The online version of this article (doi:10.1186/s12867-017-0094-z) contains supplementary material, which is available to authorized users.

## Background

Although genomic DNA usually adopts the canonical double helix structure [1–3], formation of non-canonical DNA structures, such as G-quadruplex (G4) DNA and i-motif, have also been found in human genomic DNA [2, 4, 5]. G4 is a DNA secondary structure that consists of two or more planar guanine tetrads and is stabilized by Hoogsteen hydrogen bonds along with a monovalent cation [3, 6–10]. DNA strand orientation and length of the loop sequences connecting the guanine runs provide a structural diversity to G4 forms, such as parallel, antiparallel, or a mixed structure [2, 9–12]. The characteristic topologies and existence of G4 in significant locations, such as in the telomeres and several promoters, suggest potential functions of G4 structures in gene regulation [13].

G4-forming sequences in promoter regions have been found to play a functional role in the suppression of proto-oncogenes [2, 14], such as *c*-*MYC* [15–18], *BCL*-*2* [19], *VEGF* [20], and *RET* [21]. Several studies on the G4-forming sequence within the nuclease hypersensitivity element III_1_ (NHEIII_1_) of the *c*-*MYC* promoter have reported that mutating the G4-forming sequence destabilizes the G4 structure, resulting in a three-fold increase in basal transcriptional activity of the *c*-*MYC* promoter [15, 16]. Binding of the *c*-*MYC* G4 to the cationic porphyrin TMPyP4 resulted in repression of the promoter activity [17]. Nucleolin has been identified as the *c*-*MYC* G4 binding protein that represses *c*-*MYC* expression [18]. The *BCL*-*2* gene contains a GC-rich region upstream of the P1 promoter that has been shown to be critically involved in the regulation of the *BCL*-*2* gene expression. It has been demonstrated that three individual G4 structures can be formed in this GC-rich region [19, 22]. As for *VEGF*, the stabilization of G4 DNA by quindoline derivatives represses gene transcription and consequently causes angiogenesis inhibition [20]. In addition, G4 ligands, such as TMPyP4 and telomestatin, have been shown to stabilize the G4 structure of the *RET* proto-oncogene promoter and lead to the repression of gene expression [21].

On the other hand, there are few examples of the transcriptional activation by G4 formation. A recent study of p32 G4 structure that is located in the P1 promoter of the *Bcl*-2 gene has demonstrated that reduced transcription activity in the mutated vectors compared to the native vectors [23]. This result implicated a transcriptional activation by the G4 structure. Furthermore, in the insulin-linked polymorphic region (ILPR), G4 DNA is formed by a two-repeats of consensus sequence [24, 25], and the G-quartet formation was observed to activate transcription, where single/double mutation in the sequence has reduced promoter activity [26–28]. In addition, the G4-forming *c*-*myb* GGA repeat region has shown to play a contrast role of both a transcriptional repressor and an activator, where one or two deletions of (GGA)_4_ motifs have increased *c*-*myb* promoter activity, while the deletion of all three regions has eliminated the promoter activity [29]. The involvement of G4 structures in regulating the transcription process suggests that they may hold the key to new therapeutic approaches in numerous areas of human disease, including cancer [30].

A genome-wide in silico analysis has demonstrated that sequences with the potential to form G4 motifs are enriched near transcription start sites, in the telomeres, ribosomal DNA, immunoglobulin heavy-chain switch regions, and CpG islands (CGIs) [31–33], suggesting widespread regulatory influence of the G4 motifs [34]. In a previous study, we identified 1998 G4-forming sequences in mouse CGIs using a mouse CGI microarray with a fluorescent-labeled G4 ligand L1Cy5-7OTD [35]. Among the identified G4-forming sequences, CD spectroscopy and DMS footprinting analysis were performed on 10 randomly selected G4-forming sequences and the G4 structure formation was confirmed. In this study, our aim was to analyze the biological function of the 10 G4 DNAs the in transcriptional regulation.

## Results

### *Dele* and *Cdc6* G4-forming sequences activate reporter gene expression

The 10 G4 DNA sequences from *Jard2*, *Foxa2*, *Med4*, *Chd4*, *Ntpcr*, *Bmi1*, *Wt1*, *Sp130*, *Cdc6*, and *Dele* genes (Table 1; Additional file 1) have been cloned into the luciferase reporter vector with a minimal promoter containing a TATA-box promoter element. The sequences were 42–50 nucleotides long. The successful plasmid construction has been verified by gel shift experiments and sequencing of the reporter vector.

**Table 1:** DNA sequences used in this study

Name	Sequences (5′–3′)
*Jard2*	GTGAGGCTAGG**GGG**T**GG**TG**G**TG**G**T**GGG**GGTGAGGAAGGGAAAGAT
*Dele*	ATAGCGCCAGT**GGG**T**GGG**CTTAGATCTGGGAA**GGG**C**GGG**ACAGAG
*Foxa2*	GTCCAGGAAGGCTAGA**GG**T**GGG** **GGGG**C**GGG**TACCGGTGAAGGGAG
*Chd4*	TAAAGAGGA**GGG**T**GG**CGGTAGTGGA**GGGGGGG**GTTGGAGTTGGTT
*Ntpcr*	CTTGTGTGTC**GGG**AA**GGG** **GGGGGGGG**GAGCGTTGGAAACGCATGC
*Med4*	ACTT**GGG**TA**GG**CG**GG**CTT**GG**GAGGCTCCGTTGGACGTGGGGTCTA
*Bmi1*	CACTCTTTTT**G** **GG** **G**TT**GGG**ACT**G**A**GG**T**GG**C**GG**TCACGCGAGGATC
*Wt1*	A**G**TA**GGG**A**G**CTTT**GG**AAT**G**A**GGG**ATTAACACTTT**GGGGG**ACTTAGTC
*Sp130*	AG**GG**GTA**GG**TT**GG**GT**GG**TAAGAGGTGGTAAGCGGAGCGGCTGCTG
*Cdc6*	T**GGGG**A**GG**CT**GGG**T**GG**A**GG**ACAAAGTAGAAATAAAAATACGGAAGTAGAT

Guanine runs, which form the G-quadruplex structures, are shown in bold and mutation sites are underlined

**Fig. 1:**
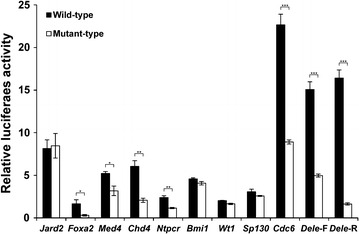
Luciferase reporter assay for evaluation of the enhancer activities of G4 DNA sequences. The G4-forming sequences were cloned into the pGL4.23 vector containing minimal promoter. *Black bars* represent the wild-types and* white bars* represent the mutant-types. Luciferase activities relative to the pGL4.23 vector are shown (mean ± SD, n = 3). Wild- and mutant-types samples t-test differences: *P < 0.01, **P < 0.001, ***P < 0.0001

The *Dele* G4-forming sequence was found on two divergently overlapping genes, *Dele* and *1700086O06Rik*, located on the mouse genome; therefore, *Dele* G4 has been cloned into the luciferase vector in the forward and reverse direction (*Dele*-F G4 and *Dele*-R G4). The reporter vectors have been transfected into NIH3T3 cells and expressed luciferase activity is measured after 48 h of cultivation. In order to reduce experimental variability the measured enzyme activity has been normalized with respect to the pGL4.74 vector which is coding for Renilla luciferase. The relative luciferase activity with and without G-quadruplex forming sequences is evaluated for a minimum of three transfections. The results of this set of experiments are compiled in Fig. 1 and show rather high expression values for *Cdc6*, *Dele*-F and *Dele*-R.

Mutant vectors have also been constructed and investigated in order to verify that really the G4-forming sequence affects the transcriptional activity. Mutant-type DNAs have been designed by replacing one of the guanine triplets to thymines (Additional file 1). These guanines were reported to be strongly involved in G4 formation [35], which can be verified by CD measurements in this study (see below).

A comparative analysis of expression levels with the G4-forming sequences and the mutant sequences demonstrates a noticeable differences for *Foxa2*, *Med4*, *Chd4*, *Ntpcr*, *Cdc6*, *Dele*-F and, *Dele*-R, but no significant difference in *Jard2*, *Bmi1*, *Wt1*, and *Sp130* (Fig. 1; Additional file 2). These results indicate that these DNA sequences possess an enhancer activity on the luciferase reporter vector, which is connected to the possibility to form specific G4 secondary structures. We focused on *Cdc6* and *Dele* G4 DNAs because the G4 DNAs showed a remarkable high activation in wild-type G4 DNAs. Moreover in regards to the statistical analysis, the t-test between wild-type and mutant-type demonstrated that *Cdc6* and *Dele* showed more significant difference (P < 0.0001) compared to *Foxa2*, *Med2* (P < 0.01) and *Chd4*, *Ntpcr* (P < 0.001); therefore, we performed detail analysis of the *Cdc6* and *Dele* G4 DNAs.

**Fig. 2:**
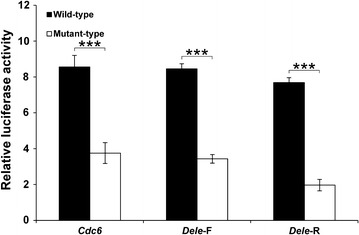
Reporter assay for evaluation of the promoter activities of *Cdc6*, *Dele*-F, and *Dele*-R G4 DNAs. The G4-forming sequences were cloned into the pGL4.10 vector not containing any promoter. *Black bars* represent the wild-types and* white bars* represent the mutant-types. Luciferase activities relative to the pGL4.10 vector are shown (mean ± SD, n = 3). Wild- and mutant-types samples t-test differences: ***P < 0.0001

It has been reported that enhancers frequently do not only interact with promoters but also promotor–promotor interaction is feasible, indicating that promoter can also work as enhancer for other gene promoters [36]. To investigate whether *Dele* and *Cdc6* G4 DNAs also possess a promoter activity, they have been cloned into a promoterless vector and then a reporter assay is performed. It is found that *Dele*-*F, Dele*-*R* and *Cdc6* G4 DNAs activate luciferase expression in this system. Furthermore, the activation of protein expression is clearly decreased by thymine mutations in the G4 region (Fig. 2; Additional file 3). These results indicate that *Dele* and *Cdc6* G4 DNAs have a role in transcriptional activation, both as a promoter and enhancer. In order to perform this function, the formation of secondary structures seems to be essential.

**Fig. 3:**
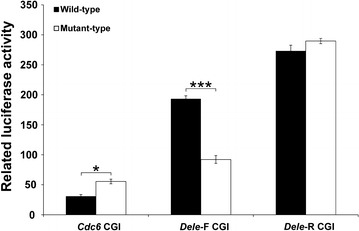
Reporter assay for evaluation of the transcriptional activity of *Cdc6*, *Dele*-F, and *Dele*-R G4 DNAs in CGI sequences. *Black bars* represent the wild-types and* white bars* represent the mutant-types. Luciferase activity relative to the pGL4.10 is shown (mean ± SD, n = 3). Wild- and mutant-types samples t-test differences: *P < 0.01, ***P < 0.0001

In order to evaluate the function of *Dele* and *Cdc6* G4 DNAs in CpG islands (CGIs) that may contain more regulatory elements, the 696-bp DNA fragment containing 475-bp *Cdc6* CGI and 555-bp DNA fragment containing 477-bp *Dele* CGI have been cloned into the promoterless vector to analyze the transcriptional activities. The results demonstrate that the luciferase activities have increased more than one order of magnitude by the cloning of *Cdc6*, *Dele*-F and *Dele*-R CGI DNAs (Fig. 3; Additional file 4). The luciferase activities are higher than that of G4 alone cloning vectors, indicating that the CGIs contain cis-regulatory elements outside of the G4 forming regions.

The transcriptional activity of the mutant-type of *Cdc6* CGI DNA has been found higher than that of the wild-type. This result suggests that *Cdc6* G4 may possess a dual function of transcriptional regulation, such as *c*-*myc* G4 [16], swinging in both ways as an activator and as a suppressor under the effect of transcriptional factors.

The transcriptional activity of the mutant-type of *Dele*-F CGI DNA has decreased compared to the wild-type. In contrast to *Dele*-F, the transcriptional activity of the *Dele*-R CGI mutant-type remains similar to that of the wild-type. Two divergently overlapping genes, *Dele* and *1700086O06Rik*, are located on the *Dele* CGI, suggesting that the *Dele* CGI may contain at least two regulatory sequences. Our results indicate that the *Dele* CGI contains a promoter for *1700086O06Rik*, an insulator, and a promoter for *Dele* (that also possesses enhancer activity). The insulator sequence may have a blocking activity that prevents promoter-enhancer interaction [37]; therefore, the enhancer activity of the *Dele* G4 for *1700086O06Rik* promoter has not been detected.

### G4 ligand suppresses the transcriptional activation of *Dele* and *Cdc6* G4 DNAs

Ligands which can bind to G-quadruplex structures can have different effect on the secondary DNA structure. Distortion or stabilization is often found depending on the binding mode of the ligand. Here the effect of a telomestatin derivative L1H1-7OTD has been investigated with respect to the effect of the G4 ligand on the transcriptional activity of the G4 DNA. This ligand can bind to the top G-tetrad structure through π-stacking and electrostatic interaction [38, 39].

**Fig. 4:**
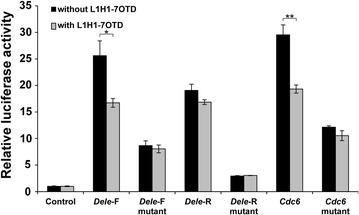
Reporter assay for evaluation of L1H1-7OTD effect on *Cdc6*, *Dele*-F, and *Dele*-R G4 DNAs. *Black bars* represent relative luciferase activities in the absence of L1H1-7OTD, and *gray bars* represent relative luciferase activities in the presence of L1H1-7OTD. Luciferase activity relative to the pGL4.23 is shown (mean ± SD, n = 3). In the presence and absence of the ligand samples t-test differences: *P < 0.01, **P < 0.001

In a first set of experiments, reporter assays with *Dele*-F, *Dele*-R and *Cdc6* G4 DNA reporter vectors carrying a minimal promoter have been performed in the presence of L1H1-7OTD. The vectors have been transfected into NIH3T3 cells and then the medium has been changed to the medium containing the G4 ligand 1 day after the transfection. After the 1 day cultivation, the luciferase activity has been measured. In this assay, results with L1H1-7OTD-treated and -untreated cells are normalized with L1H1-7OTD-treated and -untreated controls, respectively. As a result, there has been no significant difference of luciferase activity in the presence and the absence of the ligand (Additional file 5). One reason for this could be that the G4 ligand can not easily enter the cells. Therefore in a second set of experiments, the vectors have been mixed with the G4 ligand first and then transfected into NIH3T3 cells in the presence of the G4 ligand in the medium. The results show an inhibition of the luciferase expression with the wild-types of the G4 ligand-treated *Dele*-*F* and *Cdc6* G4 DNAs, whereas no significant changes have been detected with all the mutant vectors (Fig. 4; Additional file 6). Although CD measurements show a stabilizing effect of the L1H1-7OTD binding to the G quadruplex (see below), this has no beneficial effect on the activation of protein expression. In this moment one can only hypothesize that the ligand binding to the top of the G4 structure modifies the interaction of the DNA structure with respective transcription factors by influencing the dynamics, the charge and the steric conditions.

### CD spectroscopy analyses of *Dele* and *Cdc6* G4 structures

**Fig. 5:**
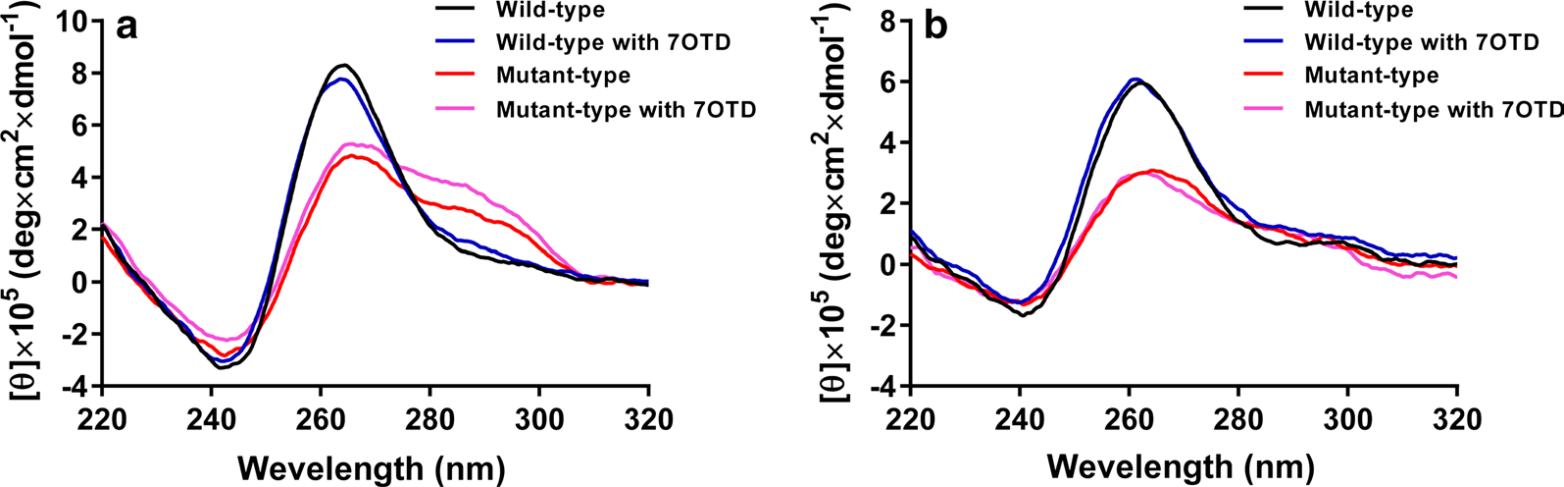
CD spectra of the *Dele* G4 (**a**) and *Cdc6* G4 DNAs (**b**). The wild-type (*black*), mutant-type (*red*), wild-type with L1H1-7OTD (*blue*), and mutant-type with L1H1-7OTD (*pink*) were analyzed at 25 °C in TK buffer (50 mM Tris–HCl, 100 mM KCl, pH 7.5)

CD spectra analysis has been performed to confirm the secondary structure formation of the wild- and mutant-type of *Dele* and *Cdc6* G4 DNA (Fig. 5; Additional file 7). The CD spectra of the wild-types show a positive cotton effect at around 264 nm for *Dele* G4 DNA, and at around 262 nm for *Cdc6* G4 DNA, with a negative effect at around 242 nm for *Dele* G4 DNA, and at around 240 nm for *Cdc6* G4 DNA. Thus, the G quadruplex formation can be confirmed under these experimental conditions.

**Fig. 6:**
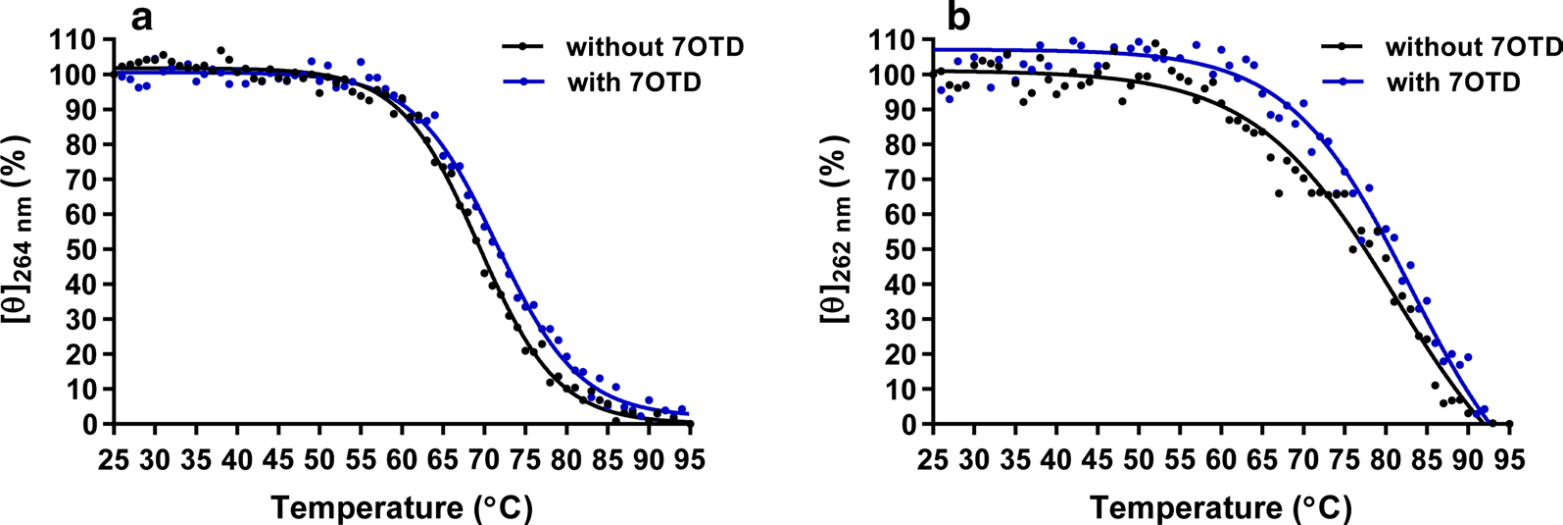
Circular dichroism melting curves of wild-types of *Dele* G4 (**a**) and *Cdc6* G4 DNAs (**b**) at 264 and 262 nm, respectively, with (*blue*) and without the ligand (*black*) in TK buffer (50 mM Tris–HCl, 100 mM KCl, pH 7.5)

In the presence of the G4 ligand (L1H1-7OTD), these spectra do not change much, indicating that the formation of parallel-type G4 structures in the wild-types of *Dele* and *Cdc6* G4 DNAs is not disturbed by the presence of the ligand (Additional files 8, 9) [40]. CD melting analysis reveals that the *T*_m_ values are 70 °C for *Dele* G4 DNA and 77 °C for *Cdc6* G4 DNA in the absence of the G4 ligand, while the *T*_m_ values increase to 72 °C for *Dele* G4 DNA, and to 81 °C for *Cdc6* G4 DNA by addition of the G4 ligand. These are clear arguments that the ligand binding to the G4 structure of the *Dele* and *Cdc6* increases the stability of the G4s at least under the chosen experimental conditions (Fig. 6; Additional file 10). The findings are in agreement with an NMR study on a structurally similar macrocyclic compound L2H2-6M(2)OTD, which has been found to bind to the top G-tetrad structure through π-stacking and electrostatic interaction. Here the folding topology did not change upon ligand binding [41].

CD spectra of the mutant-type sequences (mutating one G-run to T-run) that may form transient secondary structures, show only low molar ellipticity at around 260 nm and a shoulder between 280 and 300 nm, which are not related to the characteristic G4 structures (Fig. 5; Additional files 8, 9). Additionally in the presence of the G4 ligand, the CD spectra of the mutant-type sequences are not affected. These results strongly support the G4 formation of the two wild-type sequences of *Dele* and *Cdc6* and also verify the disability of the selected mutants to form such defined secondary structures. In consequence, the conclusions, which have been taken from the comparative analysis of mutated and wild type sequences, seem to be valid.

## Discussion

Recently, the death ligand signal enhancer (DELE) has been identified as a binding protein for the death-associated protein 3 (DAP 3), which is induced by various stimuli to regulate cell apoptosis. Stable expression of DELE induces apoptosis, whereas the knockdown of DELE has rescued HeLa cells from apoptosis induction [42]. It has also been reported that the cell-division-cycle 6 (CDC6) protein is essential for DNA replication, and the down-regulation of the *Cdc6* gene causes inhibition in cell growth accompanied by an increase in cell apoptosis [43]. Abnormal apoptosis is related to many diseases involving atrophy [44], such as Parkinson’s disease [45], with excessive cell death leading to tissue and organ damage. In contrast, some oncogenic mutations disrupt apoptosis, leading to tumor progression [46] or metastasis. Therefore, further analysis of *Dele* and *Cdc6* G4 DNAs may contribute to elucidate the apoptosis mechanism.

## Conclusions

The reporter assay for G4-forming DNA sequences has demonstrated that *Dele* and *Cdc6* G4 DNA may have the role of promoter and enhancer i.e., activating the transcription. This can be concluded from comparative experiments with mutant DNA structures. CD spectra verify the G4 formation of the wild type sequences and the disability of the studied mutants to form this secondary structure. The activation of transcription is inhibited by the telomestatin derivative L1H1-7OTD. CD spectra analysis demonstrates that binding of L1H1-7OTD stabilizes the G4 structures, but will also influence interaction with transcription factors. While for *Dele*-*F* G4 DNA transcriptional activation has also been verified in experiments with this sequence in CpG islands (CGIs), the *Cdc6* G4 DNA results here in suppression of protein expression. This indicates that *Cdc6* G4 may perform a dual functional role in the transcriptional regulation. In summary the results obtained suggest that *Dele* and *Cdc6* G4 structures are formed under physiological conditions in the cell and play a role in regulating transcription. Consequently, the study contributes to the elucidation of mechanisms of *Dele* and *Cdc6* gene regulation.

## Methods

### Plasmid construction

The G4 DNAs and mutant-type DNAs (Additional file 1) were cloned in the *Sfi*I site of the pGL4.23 [luc2/minP] or pGL4.10 [luc2] (Promega Corporation, Madison, WI, USA) and then transformed into *E. coli* DH5α (TOYOBO, Osaka, Japan). The plasmids were purified by PureYield Plasmid Miniprep System (Promega Corporation, Madison, WI, USA). *E. coli* HST04 *dam*−*/dcm*− (Takara, Tokyo, Japan) was transformed by the plasmids to prepare DNA methylation-free plasmids. All plasmids were sequenced using a 3730xl DNA analyzer (Thermo Fisher Scientific, Waltham, MA, USA). The *Dele* and *Cdc6* CGIs were amplified from C57BL/6 mouse genomic DNA by PCR. The PCR primers are shown in Additional file 11. The PCR products were purified by Wizard SV Gel and PCR Clean-Up System (Promega Corporation, Madison, WI, USA) and then digested by *Sfi*I (NEB, Ipswich, MA, USA). The products were cloned in the *Sfi*I site of pGL4.10 [luc2] and the plasmids were prepared as described above. To construct the mutant-types vectors, the site-directed mutagenesis was performed using KOD -plus- mutagenesis kit (TOYOBO, Osaka, Japan) according to manufacturer’s protocol (Additional file 11).

### Cell culture

NIH3T3 cells (RCB1862, RIKEN BRC) were cultured in dulbecco modified eagle medium (DMEM) medium (Sigma-Aldrich, St. Louis, Missouri, USA) containing 10% fetal bovine serum (Sigma-Aldrich, St. Louis, Missouri, USA), 1× penicillin–streptomycin-l-glutamine solution (Wako, Tokyo, Japan) at 37 °C in 5% CO_2_.

### Reporter assay

The NIH3T3 cells were transfected with 100 ng of the firefly luciferase reporter vector and 100 ng of the Renilla luciferase control vector by Lipofectamine 3000 (Thermo Fisher Scientific, Waltham, MA, USA) according to the manufacturer’s protocol. After 48 h, cells were harvested, and luciferase activities were measured using the Dual-Luciferase Reporter Assay System (Promega Corporation, Madison, WI, USA) and SPARK 10 M microplate reader (TECAN, Männedorf, Switzerland). The ratio of firefly luciferase activity to Renilla luciferase activity was utilized to calculate the firefly luciferase expression level in each cell and then these results were normalized with that of the pGL4.10 or pGL4.23 luciferase reporter vector. All reporter assays were performed in triplicate.

In the experiment to analyze the G4 ligand effect, two methods were used. In the first method, the 100 ng of the firefly luciferase reporter vector, and 100 ng of the Renilla control vector were transfected into NIH3T3 cells by Lipofectamine 3000. After 24 h, the medium was changed to one with 10 µM G4 ligand L1H1-7OTD. After 24 h, the luciferase activity was measured as described above. In the second method, the culture medium was changed to one with 10 µM G4 ligand L1H1-7OTD prior to transfection. The NIH3T3 cells were transfected with 100 ng of the firefly luciferase reporter vector, 100 ng of the Renilla control vector, and 10 µM of the G4 ligand L1H1-7OTD by Lipofectamine 3000, after which the luciferase activity was measured as described above. The luciferase expression level in the presence or absence of L1H1-7OTD was normalized with that of the pGL4.23 luciferase reporter vector in the presence or absence of L1H1-7OTD, respectively. All reporter assays were performed in triplicate.

### CD spectra measurement

Wild-types and mutant-types of the *Dele* and *Cdc6* G4 oligonucleotides were purchased from Macrogen, South Korea (Additional file 7). The oligonucleotides were dissolved as to create stock solutions (100 µM) in distilled water. Prior to use, all oligonucleotides were diluted to 15 µM in TK buffer (50 mM Tris–HCl, 100 mM KCl, pH 7.5). The oligonucleotides were denatured at 95 °C for 3 min and then allowed to cool to room temperature for 30 min. After heat-treatment, the oligonucleotides were diluted to 10 µM in the presence or absence of 10 µM L1H1-7OTD, and then incubated for 10 min before measurement. The CD spectra were measured with a J-1500 CD Spectrometer (JASCO, Tokyo, Japan) at 220 to 320 nm using a 1 mm path-length cuvette at every 5 °C from 20 to 95 °C. The baseline of each spectrum was corrected for signal contributions by the buffer with and without the G4 ligand. In the CD melting analysis, molar ellipticities were measured concomitantly at 1 °C intervals for wild-types of *Dele* and *Cdc6* G4 DNAs, at 264 nm and 262 nm, respectively. To determine *T*_m_ values, the molar ellipticity at 25 °C was set as 100%, and the molar ellipticity at 95 °C was set as 0%. Using GhraphPad Prism7 software, curve fitting was performed to yield normalized molar ellipticities. *T*_m_ values were recorded as temperatures equivalent to 50% of the normalized molar ellipticity.

## Additional files

**Additional file 1.** G4 DNA sequences used in the reporter assay.

**Additional file 2.** Luciferase reporter assay results of G4 DNA sequences cloned in pGL4.23 vector.

**Additional file 3.** Luciferase reporter assay results of G4 DNA sequences cloned in pGL4.10 vector.

**Additional file 4.** Luciferase reporter assay results of CGI DNA sequences cloned in pGL4.10 vector.

**Additional file 5.** Luciferase reporter assay results with or without L1H1-7OTD for G4 DNA sequences cloned in pGL4.23 vector.

**Additional file 6.** Luciferase reporter assay results with or without L1H1-7OTD for G4 DNA sequences cloned in pGL4.23 vector.

**Additional file 7.** G4 DNA sequences used in the CD Spectroscopy.

**Additional file 8.** CD spectra results of *Dele* G4 DNA wild or mutant-type sequences with or without L1H1-7OTD.

**Additional file 9.** CD spectra results of *Cdc6* G4 DNA wild-type sequence with or without L1H1-7OTD.

**Additional file 10.** Detailed molar ellipticity of wild-types of *Dele* and *Cdc6* G4 DNAs in the absence and the presence of L1H1-7OTD.

**Additional file 11.** Primer sequences used for CGI vectors construction and mutagenesis.

## Abbreviations

7OTD: 7 oxazole telomestatin derivative
CD: circular dichroism
Cdc6: cell-division-cycle 6
CpG: cytosine-phosphate-guanine
Dele: death ligand signal enhancer
G4: G-quadruplex
