# Supplementary material for: Identification of G-quadruplex structures that possess transcriptional regulating functions in the Dele and Cdc6 CpG islands
Source: BMC Mol Biol. 2017 Jun 27;18:17. doi: 10.1186/s12867-017-0094-z (PMC5488298; doi:10.1186/s12867-017-0094-z)
Supplement: Supplementary file 1 — Additional file 1. G4 DNA sequences used in the reporter assay. [file 12867_2017_94_MOESM1_ESM.docx]

**Additional file 1. G4 DNA sequences used in the reporter assay**

| Name | Sequences | | |
| --- | --- | --- | --- |
| Jard2 | 5’ | GTGAGGCTAGGGGGTGGTGGTGGTGGGGGTGAGGAAGGGAAAGAT CGG  TTG CACTCCGATCCCCCACCACCACCACCCCCACTCCTTCCCTTTCTA | 3’ |
|  | 3’ |  | 5’ |
| Jard2 MT | 5’ | GTGAGGCTAGGGGGTGGTGGTGGTTTTGGTGAGGAAGGGAAAGAT CGG  TTG CACTCCGATCCCCCACCACCACCAAAACCACTCCTTCCCTTTCTA | 3’ |
|  | 3’ |  | 5’ |
| Dele-Forward | 5’ | ATAGCGCCAGTGGGTGGGCTTAGATCTGGGAAGGGCGGGACAGAG CGG  TTG TATCGCGGTCACCCACCCGAATCTAGACCCTTCCCGCCCTGTCTC | 3’ |
|  | 3’ |  | 5’ |
| Dele-Forward MT | 5’ | ATAGCGCCAGTGGGTGGGCTTAGATCTGGGAATTTCGGGACAGAG CGG  TTG TATCGCGGTCACCCACCCGAATCTAGACCCTTAAAGCCCTGTCTC | 3’ |
|  | 3’ |  | 5’ |
| Dele-Reverse | 5’ | CTCTGTCCCGCCCTTCCCAGATCTAAGCCCACCCACTGGCGCTAT CGG  TTG GAGACAGGGCGGGAAGGGTCTAGATTCGGGTGGGTGACCGCGATA | 3’ |
|  | 3’ |  | 5’ |
| Dele-Reverse MT | 5’ | CTCTGTCCCGAAATTCCCAGATCTAAGCCCACCCACTGGCGCTAT CGG  TTG GAGACAGGGCTTTAAGGGTCTAGATTCGGGTGGGTGACCGCGATA | 3’ |
|  | 3’ |  | 5’ |
| Foxa2 | 5’ | CTCCCTTCACCGGTACCCGCCCCCCCACCTCTAGCCTTCCTGGAC CGG  TTG GAGGGAAGTGGCCATGGGCGGGGGGGTGGAGATCGGAAGGACCTG | 3’ |
|  | 3’ |  | 5’ |
| Foxa2 MT | 5’ | CTCCCTTCACCGGTACCCGCCCCAAAACCTCTAGCCTTCCTGGAC CGG  TTG GAGGGAAGTGGCCATGGGCGGGGTTTTGGAGATCGGAAGGACCTG | 3’ |
|  | 3’ |  | 5’ |
| Chd4 | 5’ | TAAAGAGGAGGGTGGCGGTAGTGGAGGGGGGGGTTGGAGTTGGTT CGG  TTG ATTTCTCCTCCCACCGCCATCACCTCCCCCCCCAACCTCAACCAA | 3’ |
|  | 3’ |  | 5’ |
| Chd4 MT | 5’ | TAAAGAGGATTTTGGCGGTAGTGGAGGGGGGGGTTGGAGTTGGTT CGG  TTG ATTTCTCCTAAAACCGCCATCACCTCCCCCCCCAACCTCAACCAA | 3’ |
|  | 3’ |  | 5’ |
| Ntpcr | 5’ | CTTGTGTGTCGGGAAGGGGGGGGGGGGAGCGTTGGAAACGCATGC CGG  TTG GAACACACAGCCCAACCCCCCCCCCCCTCGCAACCTTTGCGTACG | 3’ |
|  | 3’ |  | 5’ |
| Ntpcr MT | 5’ | CTTGTGTGTCGGGAATTTGGGGGGGGGAGCGTTGGAAACGCATGC CGG  TTG GAACACACAGCCCAAAAACCCCCCCCCTCGCAACCTTTGCGTACG | 3’ |
|  | 3’ |  | 5’ |
| Med4 | 5’ | ACTTGGGTAGGCGGGCTTGGGAGGCTCCGTTGGACGTGGGGTCTA CGG  TTG TGAACCCATCCGCCCGAACCCTCCGAGGCAACCTGCACCCCAGAT | 3’ |
|  | 3’ |  | 5’ |
| Med4 MT | 5’ | ACTTGGGTATTCGGGCTTGGGAGGCTCCGTTGGACGTGGGGTCTA CGG  TTG TGAACCCATAAGCCCGAACCCTCCGAGGCAACCTGCACCCCAGAT | 3’ |
|  | 3’ |  | 5’ |
| Bmi1 | 5’ | CACTCTTTTTGGGGTTGGGACTGAGGTGGCGGTCACGCGAGGATC CGG  TTG GTGAGAAAAACCCCAACCCTGACTCCACCGCCAGTGCGCTCCTAG | 3’ |
|  | 3’ |  | 5’ |
| Bmi1 MT | 5’ | CACTCTTTTTGTTGTTGGGACTGAGGTGGCGGTCACGCGAGGATC CGG  TTG GTGAGAAAAACAACAACCCTGACTCCACCGCCAGTGCGCTCCTAG | 3’ |
|  | 3’ |  | 5’ |
| Wt1 | 5’ | AGTAGGGAGCTTTGGAATGAGGGATTAACACTTTGGGGGACTTAGTC CGG  TTG TCATCCCTCGAAACCTTACTCCCTAATTGTGAAACCCCCTGAATCAG | 3’ |
|  | 3’ |  | 5’ |
| Wt1 MT | 5’ | AGTATTTAGCTTTGGAATGAGGGATTAACACTTTGGGGGACTTAGTC CGG  TTG TCATAAATCGAAACCTTACTCCCTAATTGTGAAACCCCCTGAATCAG | 3’ |
|  | 3’ |  | 5’ |
| Sp130 | 5’ | AGGGGTAGGTTGGGTGGTAAGAGGTGGTAAGCGGAGCGGCTGCTG CGG  TTG TCCCCATCCAACCCACCATTCTCCACCATTCGCCTCGCCGACGAC | 3’ |
|  | 3’ |  | 5’ |
| Sp130 MT | 5’ | AGGGGTAGGTTTTGTGGTAAGAGGTGGTAAGCGGAGCGGCTGCTG CGG  TTG TCCCCATCCAAAACACCATTCTCCACCATTCGCCTCGCCGACGAC | 3’ |
|  | 3’ |  | 5’ |
| Cdc6 | 5’ | TGGGGAGGCTGGGTGGAGGACAAAGTAGAAATAAAAATACGGAAGTAGAT CGG  TTG ACCCCTCCGACCCACCTCCTGTTTCATCTTTATTTTTATGCCTTCATCTA | 3’ |
|  | 3’ |  | 5’ |
| Cdc6 MT | 5’ | TGGGGAGGCTTTTTGGAGGACAAAGTAGAAATAAAAATACGGAAGTAGAT CGG  TTG ACCCCTCCGAAAAACCTCCTGTTTCATCTTTATTTTTATGCCTTCATCTA | 3’ |
|  | 3’ |  | 5’ |

Guanine runs, which form the G-quadruplex structures, are shown in red and mutation sites are highlighted.
