# Supplementary material for: Identification of G-quadruplex structures that possess transcriptional regulating functions in the Dele and Cdc6 CpG islands
Source: BMC Mol Biol. 2017 Jun 27;18:17. doi: 10.1186/s12867-017-0094-z (PMC5488298; doi:10.1186/s12867-017-0094-z)
Supplement: Supplementary file 7 — Additional file 7. G4 DNA sequences used in the CD Spectroscopy. [file 12867_2017_94_MOESM7_ESM.docx]

**Additional file7. G4 DNA sequences used in the CD Spectroscopy**

| Name | Sequences |
| --- | --- |
| Mouse *Dele* G4 | 5’-GGGTGGGCTTAGATCTGGGAAGGGCGGG-3’ |
| Mouse *Dele* G4 MT | 5’-GGGTGGGCTTAGATCTGGGAA**TTT**CGGG-3’ |
| Mouse *Cdc6* G4 | 5’-GGGGAGGCTGGGTGGAGG-3’ |
| Mouse *Cdc6* G4 MT | 5’-GGGGAGGCT**TTT**TGGAGG-3’ |

Mutation sites are shown in bold.
