# Supplementary material for: Identification of G-quadruplex structures that possess transcriptional regulating functions in the Dele and Cdc6 CpG islands
Source: BMC Mol Biol. 2017 Jun 27;18:17. doi: 10.1186/s12867-017-0094-z (PMC5488298; doi:10.1186/s12867-017-0094-z)
Supplement: Supplementary file 11 — Additional file 11. Primer sequences used for CGI vectors construction and mutagenesis. [file 12867_2017_94_MOESM11_ESM.docx]

**Additional file 11. Primer sequences used for CGI vectors construction and mutagenesis**

| Primer |  | Sequences |
| --- | --- | --- |
| *Dele*-F CGI | Forward | 5’-ATTGGCCTAACTGGCCAGGGTGCTCTAGGTTCACCA-3’ |
|  | Reverse | 5’-ATTGGCCGCCGAGGCCTCCCCTTGGACCTAAGCTCT-3’ |
| *Dele*-R CGI | Forward | 5’-ATTGGCCTAACTGGCCTCCCCTTGGACCTAAGCTCT-3’ |
|  | Reverse | 5’-ATTGGCCGCCGAGGCCAGGGTGCTCTAGGTTCACCA-3’ |
| *Cdc5* CGI | Forward | 5’-ATTGGCCTAACTGGCCATGAGCAAAGGTAGCCCAGT-3’ |
|  | Reverse | 5’-ATTGGCCGCCGAGGCCCTGCTCAAAACTAGCCAGCA-3’ |
| *Dele* CGI MT | Forward | 5’-CGGGACAGAGGGAGCGAGG-3’ |
|  | Reverse | 5’-**AAA**TTCCCAGATCTAAGCCCACCCACT-3’ |
| *Cdc5* CGI MT | Forward | 5’-TGGAGGACAAAGTAGAAATAAAAATACG-3’ |
|  | Reverse | 5’-**AAA**AGCCTCCCCACCGTTGCC-3’ |

Mutation sites are shown in bold.
